# Migraine with comorbid fibromyalgia: psychological burden and impact on frequency and severity of migraine attacks

**DOI:** 10.3389/fneur.2026.1776543

**Published:** 2026-04-30

**Authors:** Amr Hassan, Mona Hussein, Rehab Magdy, Anas Elgenidi, Nahla Merghany, Ahmed Yehia Ismaeel, Mohamed Abdelghaffar, Osama Yacoub, May M. Fayez, Nourhan Abdelmohsen Taha, Ramez Reda Moustafa, Ahmed Essmat

**Affiliations:** 1 Department of Neurology, Cairo University, Cairo, Egypt; 2 Department of Neurology, Beni-Suef University, Beni-Suef, Egypt; 3 Department of Neurology, Mayo Clinic, Jacksonville, FL, United States; 4 Department of Internal Medicine, Beni-Suef University, Beni-Suef, Egypt; 5 Department of Neurology, Fayoum University, Fayoum, Egypt; 6 Department of Neurology, El Mounira Hospital, Cairo, Egypt; 7 Department of Neurology, Ain Shams University, Cairo, Egypt; 8 Department of Neurology, Al Azhar University, Cairo, Egypt

**Keywords:** DASS-12, fibromyalgia, FIQR, HIT-6, migraine, psychological burden

## Abstract

**Background:**

There is growing evidence suggesting a bidirectional relationship between fibromyalgia and migraine. The aim of this work was to study the impact of fibromyalgia on the frequency and severity of migraine headache attacks and the psychological burden in patients with comorbid migraine and fibromyalgia.

**Methods:**

This cross-sectional comparative study was conducted on 114 patients with comorbid migraine and fibromyalgia and 270 patients with migraine only. All included patients were subjected to a comprehensive assessment of headache through face-to-face interview and assessment of the psychological state using the Depression, Anxiety and Stress Scale-12 (DASS-12). Patients with fibromyalgia were requested to answer the Revised Fibromyalgia Impact Questionnaire (FIQR).

**Results:**

Patients with comorbid migraine and fibromyalgia had significantly higher monthly migraine days (MMD), headache impact test-6 (HIT-6), and DASS-12 total scores than patients with migraine only (*p*-value < 0.001, 0.009, and < 0.001, respectively). There was a statistically significant positive correlation between the FIQR total score and DASS-12 total score (*p*-values = 0.001). Additionally, statistically significant positive correlations were found between the FIQR total score and both the MMD and HIT-6 total scores (*p*-values = 0.002 and 0.008, respectively). There were also statistically significant positive correlations between the DASS-12 total score and both the MMD and HIT-6 total scores (*p*-values = 0.006, 0.027, respectively).

**Conclusion:**

Fibromyalgia is an aggravating comorbid condition with migraine that is associated with a higher frequency and severity of migraine headache attacks, in addition to increasing the psychological burden.

## 1 Introduction

Migraine is a complex neurological condition characterized by recurrent episodes of moderate to severe headaches that affect daily life. It affects approximately 15.2% of the general population According to the 2021 estimates of the Global Burden of Disease study (GBD) (1–3) This prevalence is reportedly higher in Egypt, with 20.9% of the general population (4). The presence of specific comorbidities like anxiety and depression, and clinical conditions like epilepsy and cardiovascular diseases, contributes to a higher prevalence (5).

Recent literature reports that patients with fibromyalgia report a higher frequency of severe headaches in addition to poor quality of life (6). Fibromyalgia is a clinical condition characterized by generalized musculoskeletal pain and multiple tender points, with a prevalence of 13.2% in the Egyptian population (7, 8). Benn et al. reported the bidirectional relationship of both fibromyalgia and migraine that tends to coexist and can contribute to increased disability and severity (6).

There is growing evidence suggesting a familial co-occurrence of these two conditions (9). Central somatization, in which the brain becomes more sensitive to stimuli, and disturbances in neurotransmitters, such as high glutamate levels, are considered shared pathophysiologic mechanisms involved in both fibromyalgia and migraine (10). The presence of severe depressive symptoms and severe headache, which has a poor response to treatment in patients with migraine, may raise concern for screening for comorbid fibromyalgia (11).

Furthermore, accurate diagnosis of this comorbidity has crucial implications for the management of these patients through a multidisciplinary approach, including both pharmacological and nonpharmacological strategies. Preferred treatments aim to target common pathways, such as anti-Calcitonin gene-related peptide (CGRP) monoclonal antibodies. Future therapies may focus on the transient receptor potential ankyrin 1 (TRPA1) channel and glutamate receptors, which are involved in the pathophysiology of both conditions (10, 12).

Despite the high prevalence of migraine and fibromyalgia in Egypt and their crucial implications, no studies have reported the effect of fibromyalgia on the migraine characteristics and associated psychiatric symptoms. Therefore, the aim of this work was to study the impact of fibromyalgia on the frequency and severity of migraine headache attacks in patients with comorbid migraine and fibromyalgia. The second objective was to study the impact of both disorders on the psychological burden of those patients.

## 2 Materials and methods

### 2.1 Study design and participants

This cross-sectional comparative study was conducted on female patients with comorbid migraine and fibromyalgia and age and sex matched patients with migraine only. The patients were recruited in the period from March 2025 to June 2025, from four headache clinics in Cairo, Beni-Suef, El-Fayoum, and Al-Azhar University Hospitals.

**Figure 1:**
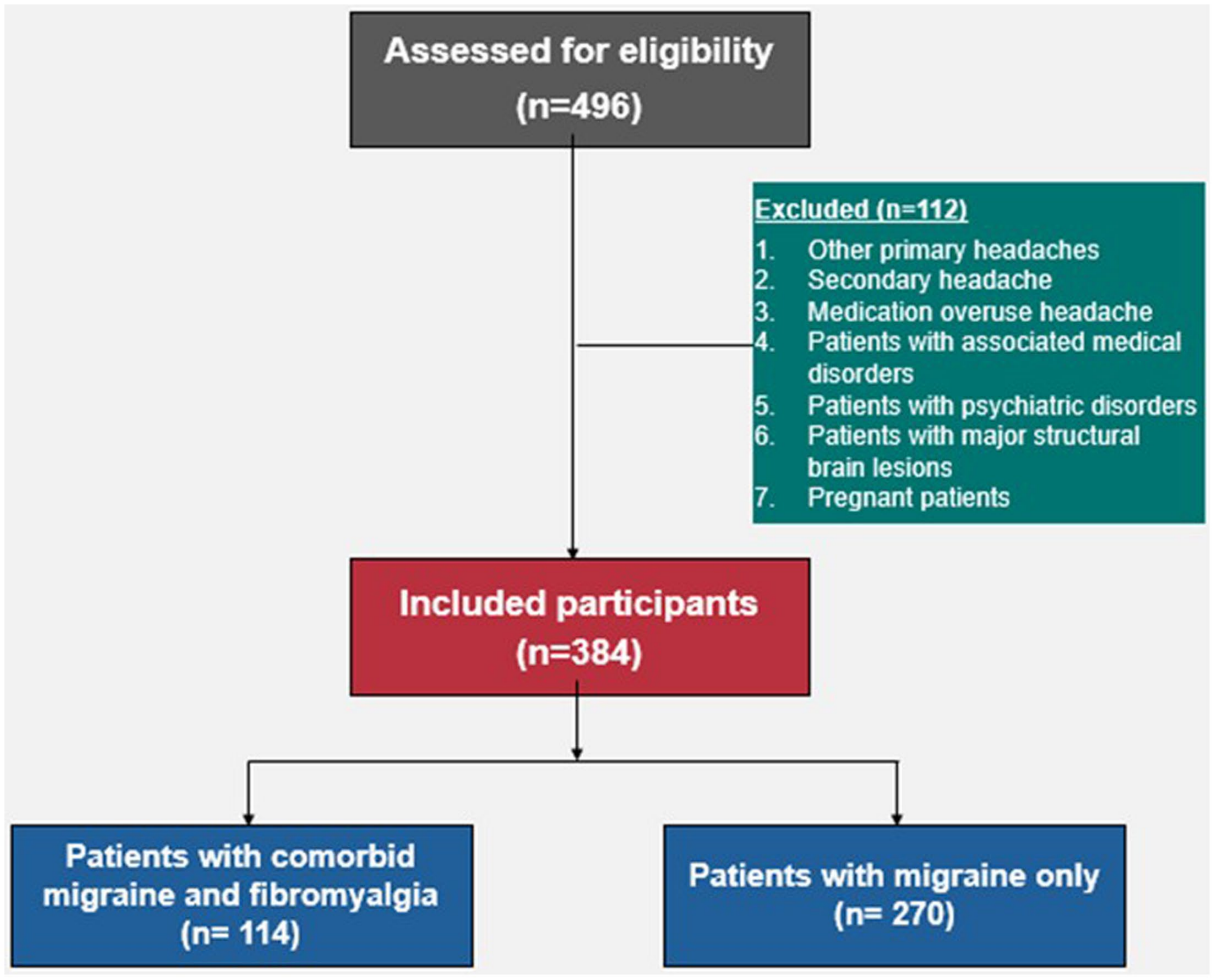
A flow diagram for the included and excluded participants.

Patients with migraine should fulfill the diagnostic criteria for migraine whether episodic or chronic according to the International Classification of Headache Disorders, 3rd edition (ICHD-3) (13). Patients with fibromyalgia should fulfill the diagnostic criteria for fibromyalgia according to the 2016 American College of Rheumatology (14). The following patients were excluded from the study: patients with other primary headaches, secondary headache, or medication overuse headache, patients with associated medical disorders (other than fibromyalgia) that might negatively impact the psychological state, patients fulfilling Diagnostic and Statistical Manual of Mental Disorders (DSM-5) criteria (15) for psychiatric disorders such as major depression, psychosis, or anxiety disorders, patients with major structural brain lesions, and pregnant patients. A flow diagram for the included and excluded participants is shown in Figure 1.

### 2.2 Clinical assessment

All included patients were subjected to a comprehensive assessment of headache through a face-to-face interview. The following data were obtained: disease duration, monthly migraine days (MMD), type of migraine, whether episodic or chronic and the presence of allodynia. Assessment of headache severity was done using the following scales:

1. Visual analogue scale (VAS) (16): It is a 10-point scale used to assess pain intensity. The patients were asked to rate their headache intensity between 1 (very mild headache) and 10 (severe, intolerable headache).
2. Headache Impact test −6 (HIT-6) (17): It is a six-item scale that rates the functions mostly affected by headache, including activities of daily living, social life, pain, attention, psychological state, and fatigue. Each item is rated using five responses (always, very often, sometimes, never, or rarely). The total HIT-6 score ranges between 36 and 78, with higher scores reflecting a severe impact of headache on the quality of life (18).

Patients with fibromyalgia were requested to answer the Fibromyalgia Impact Questionnaire Revised (FIQR) (19). The FIQR consists of 21 questions covering three domains: function, overall impact, and symptoms. Each question is rated on an 11-point numeric rating scale from 0 to 10, with ten being the worst score. The Arabic version of FIQR was used (20).

Assessment of the psychological state for all included patients was done using the Depression, Anxiety and Stress Scale-12 items (DASS-12) (21). It is a 12-item questionnaire that is used to evaluate depression (DASS-D), anxiety (DASS-A), and stress (DASS-S) over the last week. Each domain includes four items. The total score is the sum of the three domains’ subscores. It ranges from 0 to 21. Higher scores indicates worst psychological state. The Arabic version of DASS-12 was used (22). The DASS-12 was used to quantify subclinical psychological symptoms among participants without formally diagnosed psychiatric disorders.

### 2.3 Ethics statement

Written informed consents were obtained from the included participants. Data were confidential and anonymous. Ethical approval was obtained from the Scientific Research Ethics Committee, Faculty of Medicine, Fayoum University. The approval number is R716.

### 2.4 Sample size calculation

The sample size was calculated using G*Power version 3.1.9.7 Software. The effect size was calculated based on mean values of HIT-6 total scores in migraine patients with and without fibromyalgia in a study conducted by Beyazal et al. (2018) (23). The used statistical test was independent sample t-test. The type of power analysis was: *A priori*: compute required sample size- given *α*, power, and effect size. The input parameters were: tail (s) = two, effect size d = 0.385, α err prob. = 0.05, and power (1-*β* err prob) = 0.80. The output parameters were: noncentrality parameter *δ* = 2.819, critical t = 1.971, and Df = 212. A total sample size of at least 107 in each group was required to achieve a statistical power (1–β) 80%.

### 2.5 Statistical methods

Data were analyzed using Statistical Package for the Social Sciences (SPSS) version 25 (IBM Corp., Armonk, NY, USA). The Kolmogorov–Smirnov test was used to test the normality of data. Quantitative data such as age, body mass index (BMI), disease duration, VAS, MMD, HIT-6, DASS-12, and FIQR total scores were expressed as median and interquartile range (IQR). Categorical data such as sex, type of migraine, allodynia, and type of fibromyalgia were expressed as numbers and percentages. The Mann–Whitney U test was used for comparison between migraine patients with and without fibromyalgia regarding age, BMI, disease duration, VAS, MMD, HIT-6, and DASS-12. Spearman correlation was used to make correlations between VAS, MMD, HIT-6, DASS-12, and FIQR. Binary logistic regression analysis was done to identify factors independently associated with chronic migraine. Multiple linear regression analysis was done to identify factors independently associated with DASS-12 total score. *p*-value < 0.05 was considered statistically significant. All tests were two-tailed.

## 3 Results

### 3.1 Demographics and characteristics of headache attacks in migraine patients with fibromyalgia versus those without

**Figure 2:**
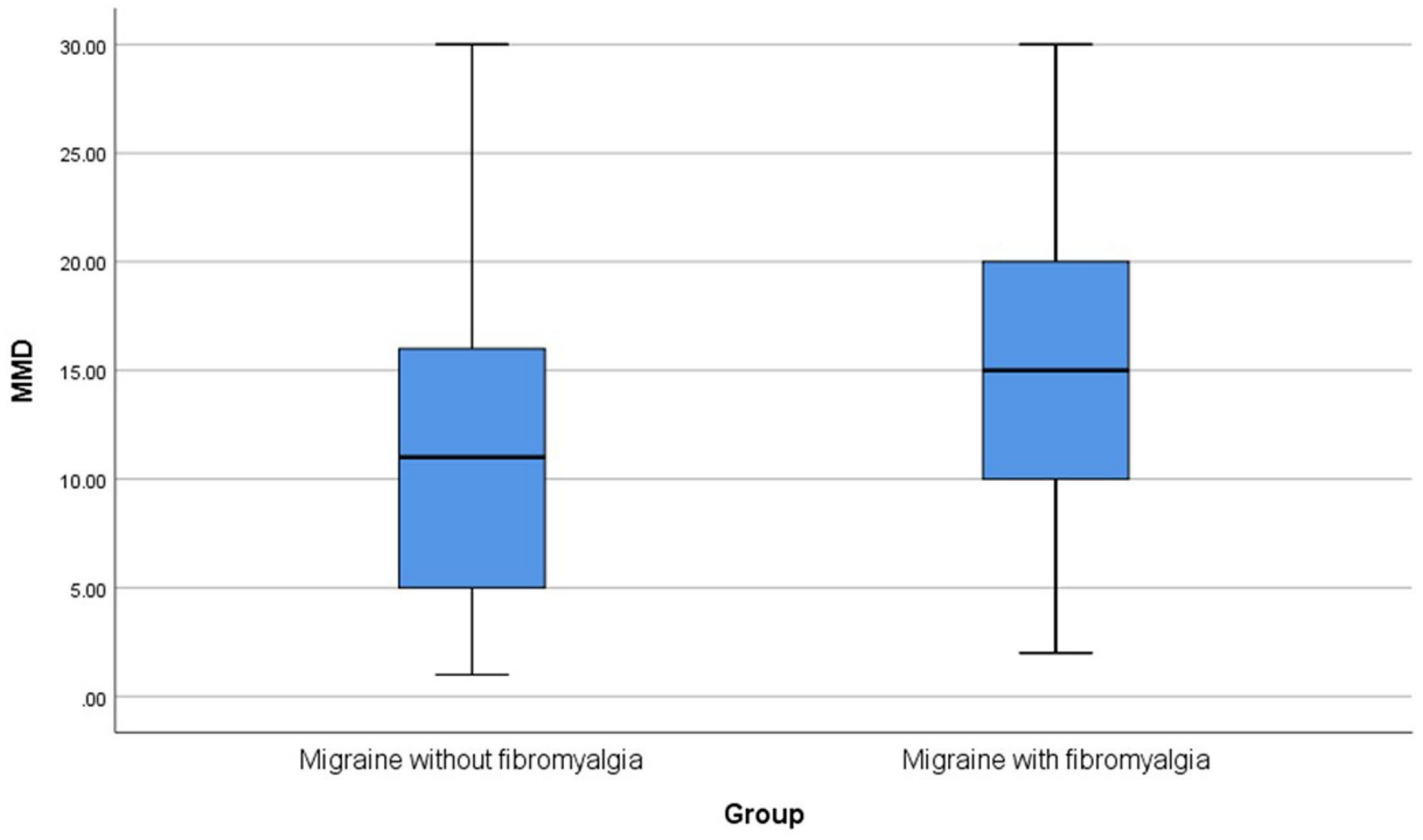
Boxplots for MMD. MMD, monthly migraine days.

**Figure 3:**
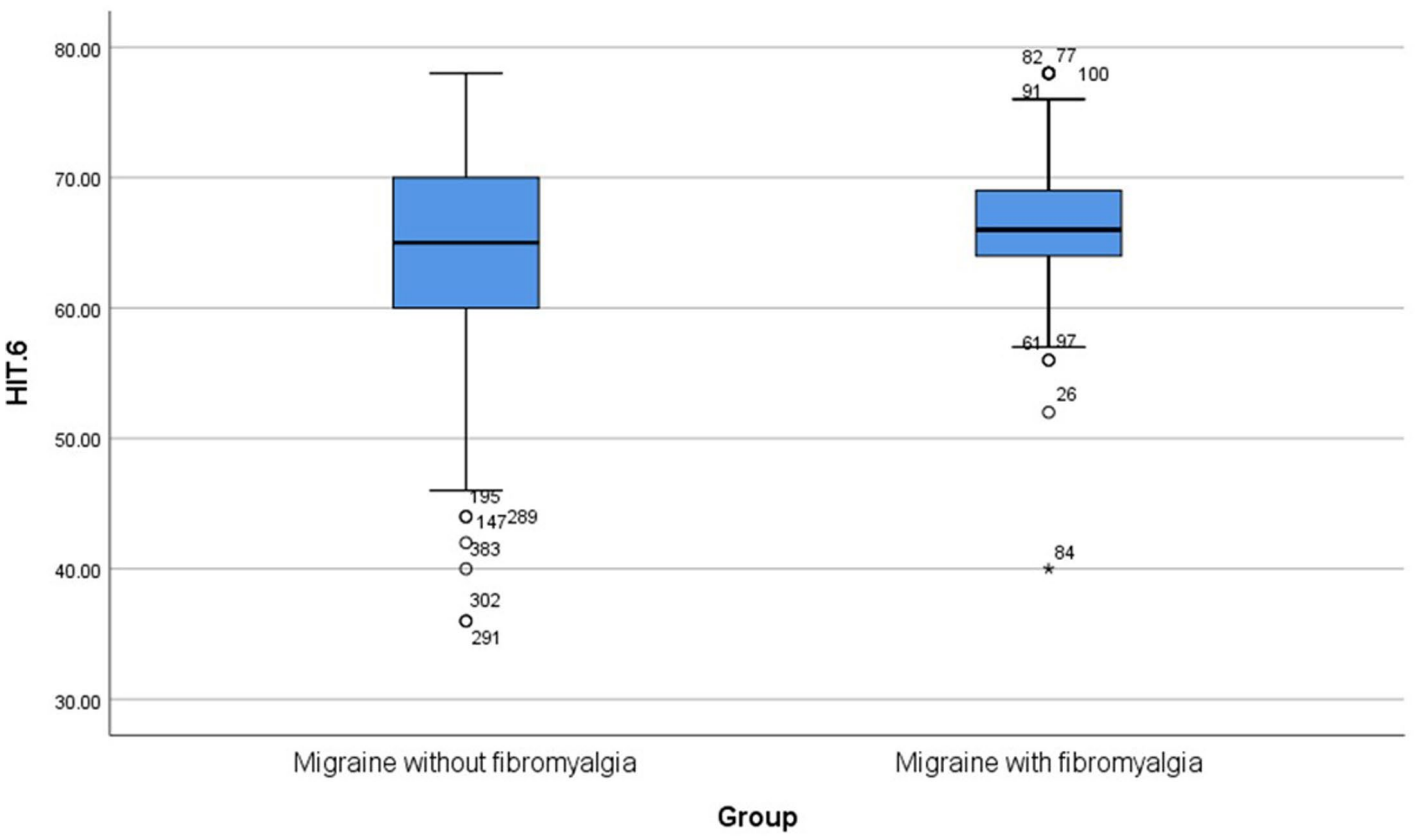
Boxplots for HIT-6. HIT-6, headache impact test-6.

This cross-sectional comparative study was conducted on 114 patients with comorbid migraine and fibromyalgia and 270 patients with migraine only. There was no statistically significant difference in age between patients with comorbid migraine and fibromyalgia and those with migraine only (*p* = 0.052). Patients with comorbid migraine and fibromyalgia had a significantly higher frequency of chronic migraine and allodynia than patients with migraine only (*p*-value < 0.001, 0.001, respectively). Also, patients with comorbid migraine and fibromyalgia had significantly higher MMD and HIT-6 total scores than patients with migraine only (*p*-value < 0.001, 0.009, respectively) (Table 1; Figures 2,3).

**Table 1:** Demographics and characteristics of headache attacks in migraine patients with fibromyalgia versus those without.

Item		Patients with comorbid migraine and fibromyalgia (*n* = 114)	Patients with migraine only (*n* = 270)	*p*-value
Age [median (IQR)]		35 (29–42)	33 (25–41)	0.052
BMI [median (IQR)]		29.56 (25.54–33.44)	27.36 (24.61–31.51)	0.003*
Disease duration in years (migraine) [median (IQR)]		7 (3.75–15)	6 (3–13)	0.087
Type of migraine	Episodic [n (%)]	36 (31.6%)	171 (63.3%)	< 0.001*
	Chronic [n (%)]	78 (68.4%)	99 (36.7%)	
Allodynia [n (%)]		93 (81.6%)	173 (64.1%)	0.001*
Intensity of migraine attacks by VAS [median (IQR)]		8 (6–9)	7 (5–9)	0.512
MMD [median (IQR)]		15 (10–20)	11 (5–16)	< 0.001*
HIT-6 total score [median (IQR)]		66 (64–69)	65 (60–70)	0.009*

BMI, body mass index; HIT-6, headache impact test-6; MMD: monthly migraine days; VAS, visual analogue scale.

^*^p-value < 0.05 is considered significant. Bold indicates statistically significant value.

### 3.2 Depression, anxiety, and stress in migraine patients with fibromyalgia versus those without

**Figure 4:**
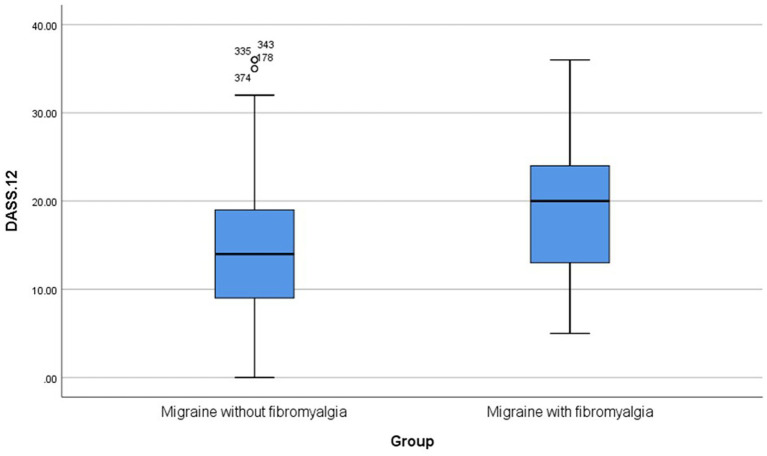
Boxplots for DASS-12. DASS-12, Depression, Anxiety, and Stress Scale-12.

Patients with comorbid migraine and fibromyalgia had significantly higher depression, anxiety, stress, and DASS-12 total score in comparison to patients with migraine only (p-value < 0.001 in all comparisons) (Table 2; Figure 4).

**Table 2:** Depression, anxiety, and stress in migraine patients with fibromyalgia versus those without.

DASS-12	Patients with comorbid migraine and fibromyalgia (*n* = 114) [median (IQR)]	Patients with migraine only (*n* = 270) [median (IQR)]	*p*-value
Depression score (DASS-D)	5.5 (3–8)	3 (1–6)	< 0.001*
Anxiety score (DASS-A)	6 (5–9)	5 (3–7)	< 0.001*
Stress score (DASS-S)	8 (5–9)	6 (4–8)	< 0.001*
DASS-12 total score	20 (12–24)	14 (9–19)	< 0.001*

DASS-12, Depression, Anxiety, and Stress Scale-12.

^*^p-value < 0.05 is considered significant. Bold indicates statistically significant value.

### 3.3 Clinical characteristics of fibromyalgia in migraine patients with comorbid fibromyalgia

The median value for disease duration was 5 years (range, 3–9 years). About 85% (*n* = 97) of patients had primary fibromyalgia, and 14.9% (*n* = 17) had secondary fibromyalgia. The median value for the function domain score was 19.33 (7.33–24.08), for the overall impact score was 14 (10–16), for the symptoms score was 40 (32–43.63), and for FIQR total score was 71.08 (55.33–81.04) (Table 3).

**Table 3:** Clinical characteristics of fibromyalgia in migraine patients with comorbid fibromyalgia.

Item		Patients with comorbid migraine and fibromyalgia (*n* = 114)
Disease duration in years (Fibromyalgia) [median (IQR)]		5 (3–9)
Type of fibromyalgia	Primary [*n* (%)]	97 (85.1%)
	Secondary [*n* (%)]	17 (14.9%)
FIQR [median (IQR)]	Domain 1: Function	19.33 (7.33–24.08)
	Domain 2: Overall impact	14 (10–16)
	Domain 3: Symptoms	40 (32–43.63)
	FIQR total score	71.08 (55.33–81.04)

FIQR: fibromyalgia impact questionnaire revised.

### 3.4 The relationship between severity of fibromyalgia, severity of depression, anxiety, and stress, and frequency and severity of migraine headache attacks

There were statistically significant positive correlations between the FIQR total score and the scores of depression, anxiety, stress, and the DASS-12 total score (*p*-values = 0.003, 0.006, 0.003, and 0.001, respectively) (Table 4).

**Table 4:** The relationship between severity of fibromyalgia and severity of depression, anxiety, and stress.

Variable	FIQR total score	
	(r) Coef.	*p*-value
Depression score (DASS-D)	0.277	0.003*
Anxiety score (DASS-A)	0.257	0.006*
Stress score (DASS-S)	0.273	0.003*
DASS-12 total score	0.320	0.001*

DASS-12, Depression, Anxiety, and Stress Scale-12; FIQR, Fibromyalgia impact questionnaire revised.

(r), Spearman correlation coefficient; *p-value < 0.05 is considered significant. Bold indicates statistically significant value.

**Figure 5:**
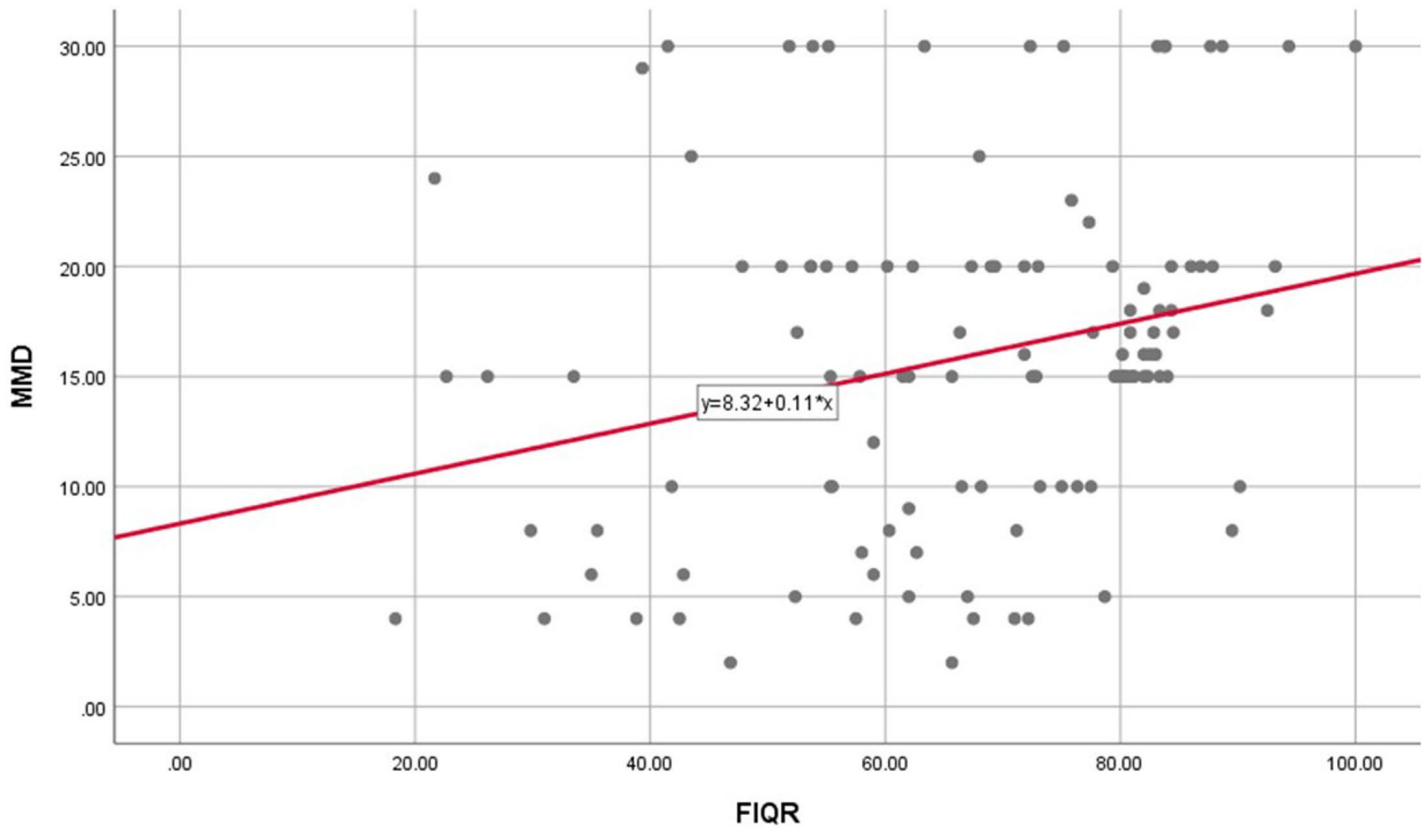
Correlations between FIQR and MMD. FIQR, fibromyalgia impact questionnaire revised; MMD, monthly migraine days.

Additionally, statistically significant positive correlations were found between the FIQR total score and both the MMD and HIT-6 total scores (*p*-values = 0.002 and 0.008, respectively). There were also statistically significant positive correlations between the DASS-12 total score and both the MMD and HIT-6 total scores (*p*-values = 0.006 and 0.027, respectively) (Table 5; Figure 5).

**Table 5:** Impact of severity of fibromyalgia, depression, anxiety, and stress on frequency and severity of migraine headache attacks.

Variable	FIQR total score		DASS-12 total score	
	(r) Coef.	*p*-value	(r) Coef.	*p*-value
MMD	0.282	0.002*	0.258	0.006*
Intensity of migraine attacks by VAS	0.150	0.112	0.148	0.116
HIT-6 total score	0.245	0.008*	0.208^*^	0.027*

DASS-12, Depression, Anxiety, and Stress Scale-12; FIQR, Fibromyalgia impact questionnaire revised; HIT-6, Headache impact test-6; MMD, Monthly migraine days; VAS, Visual analogue scale.

(r): Spearman correlation coefficient, *p-value < 0.05 is considered significant.Bold indicates statistically significant value.

### 3.5 Factors associated with chronic migraine

Binary logistic regression analysis was done to identify factors independently associated with chronic migraine. The independent variables included in the model were: age, BMI, fibromyalgia, migraine duration, allodynia, and DASS-12 total score The adjusted model revealed that fibromyalgia (OR = 2.803, 95% CI: 1.687–4.657, *p*-value < 0.001), longer migraine duration (OR = 1.033, 95% CI: 1.002–1.065, *p*-value = 0.038), allodynia (OR = 1.915, 95% CI: 1.160–3.161, *p*-value = 0.011), and higher DASS-12 total score (OR = 1.077, 95% CI: 1.045–1.110, *p*-value < 0.001) were all significantly associated with chronic migraine (Table 6).

**Table 6:** Factors associated with chronic migraine.

Variable	B	Wald Chi square	*p*-value	Odds ratio	95% C. I.for EXP(B)	
					Lower	Upper
Age	0.003	0.035	0.851	1.003	0.974	1.032
BMI	−0.040	2.827	0.093	0.961	0.918	1.007
Fibromyalgia	1.031	15.837	< 0.001*	2.803	1.687	4.657
Migraine duration	0.033	4.325	0.038*	1.033	1.002	1.065
Allodynia	0.650	6.460	0.011*	1.915	1.160	3.161
DASS-12 total score	0.074	22.721	< 0.001*	1.077	1.045	1.110
Constant	−1.383	4.098	0.043	0.251		

BMI, body mass index; DASS-12, Depression, Anxiety, and Stress Scale-12.

^*^p-value < 0.05 is considered significant.Bold indicates statistically significant value.

### 3.6 Factors associated with total score of DASS-12

Multiple linear regression analysis was done to identify factors independently associated with DASS-12 total score. The independent variables included in the model were BMI, fibromyalgia status, MMD, VAS, chronic migraine, and HIT-6 total score. The adjusted model revealed that fibromyalgia (B = 3.031, 95% CI: 1.311–4.750, *p*-value = 0.001), MMD (B = 0.178, 95% CI: 0.022–0.334, *p*-value = 0.025), VAS (B = 0.319, 95% CI: 0.025–0.613, *p*-value = 0.033), and HIT-6 total score (B = 0.169, 95% CI: 0.062–0.277, *p*-value = 0.002) were all significantly associated with higher DASS-12 total score (Table 7).

**Table 7:** Factors associated with total score of DASS-12.

Variable	B	*p*-value	95.0% CI for B		Collinearity statistics	
			Lower bound	Upper bound	Tolerance	VIF
(Constant)	−2.777	0.505	−10.959	5.404		
BMI	0.040	0.567	−0.097	0.177	0.951	1.052
Fibromyalgia	3.031	0.001*	1.311	4.750	0.880	1.136
MMD	0.178	0.025*	0.022	0.334	0.337	2.966
VAS	0.319	0.033*	0.025	0.613	0.960	1.042
Chronic migraine	0.724	0.590	−1.916	3.364	0.314	3.187
HIT-6 total score	0.169	0.002*	0.062	0.277	0.842	1.188

BMI, body mass index; HIT-6, headache impact test-6; MMD, monthly migraine days; VAS, visual analogue scale.

*p-value < 0.05 is considered significant.Bold indicates statistically significant value.

## 4 Discussion

Studying fibromyalgia as a common comorbidity with migraine and its association with headache disability can guide early, effective treatment and minimize the disease burden. Compared to those without fibromyalgia, patients with comorbid fibromyalgia had an increased frequency and intensity of headache attacks, and more intense depression and anxiety symptoms that adversely affect their quality of life. Consistent with published data (11, 24), fibromyalgia was more common in chronic migraine than in episodic type. Further, the severity of fibromyalgia symptoms contributes to the disabling of migraine attacks.

The robust association between fibromyalgia and migraine-related disability detected in the current study could be multifactorial. First, central sensitization and impaired descending pain modulation are shared by the two conditions (25, 26), may help elucidate this relationship. On the other hand, this could explain why migraine patients with fibromyalgia may exhibit allodynia, a manifestation of central sensitization (27), more significantly than those with migraine only. Yet, de Tommaso, Sardaro (24) stated that the presence of allodynia did not differ in migraine patients with and without fibromyalgia.

Also, obesity serves as an overlapping risk factor for the two disorders. Indeed, we observed that patients with comorbid migraine and fibromyalgia had a significantly higher BMI than those with migraine only. Obesity in fibromyalgia is associated with greater levels of proinflammatory markers implicated in central sensitization (28, 29).

From another perspective, the two conditions shared psychological disturbances. It was reported that patients with anxiety and depression symptoms may have a disturbed pain modulation with augmentation of central sensitization (30). It is also expected that chronic pain may boost depression and anxiety symptoms, promoting a self-perpetuating circuit that eventually builds up the pain severity (31). Such an explanation might account for why anxiety and depression symptoms significantly prevailed in patients with comorbid migraine and fibromyalgia than in those with migraine only, which is further emphasized by the significant correlations found between DASS-12 and FIQR scores. Previous reports have obtained similar results, although they used different assessment tools, including the Patient Health Questionnaire-9 (PHQ-9) by Whealy, Nanda (11), and the Zung Self-Rating Anxiety and Depression scales by de Tommaso, Sardaro (24).

The current findings indicate that fibromyalgia is significantly associated with chronic migraine (OR = 2.803), underlining the central sensitization that arises in both disorders (32). Further, allodynia, a key feature of fibromyalgia (33), has also been found to be significantly associated with chronic migraine (OR = 1.915). Additionally, the multivariate analysis revealed that the comorbidity of fibromyalgia is associated with increased risk of anxiety and depression, a well-established risk factor of chronic migraine (23). These findings underscore the systematic screening and evaluation for fibromyalgia for providing a more tailored approach to migraine management. Interestingly, comorbid fibromyalgia was also found to impact migraine management and prognosis. Through a large longitudinal study, Pensato, Ornello (34) observed a strong association between fibromyalgia and worsening migraine response to treatment.

Future studies are required to employ updated patient-reported treatment outcome measures (PROMs) and more objective measures of headache-related disability to assess treatment response, as well as to examine the inter-ictal periods during which individuals may also experience negative effects of this comorbidity (35).

### 4.1 Limitations and future recommendations

Due to the relatively small sample size, comparison between subgroups of patients was not feasible, e.g., primary versus secondary fibromyalgia. Additionally, the current study design can only establish associations, rather than causal relationships, between the two disorders. Future studies of a longitudinal design are necessary to verify the nature of the recognized association and to elucidate the underlying pathophysiology, which could pave the way for the development of optimal therapeutic approaches. It would be of clinical significance to determine whether more aggressive treatment is needed for migraine patients with comorbid fibromyalgia.

Notably, this study included only female patients. This was partly due to the use of FIQR to assess fibromyalgia severity, which was validated exclusively in female populations. Undoubtedly, this limits the generalizability of the findings, and the results cannot be directly extrapolated to male patients. Future studies including both sexes are warranted.

## 5 Conclusion

Fibromyalgia is an aggravating comorbid condition with migraine that is associated with a higher frequency and greater disability of headache attacks, as well as more symptoms of depression and anxiety, and eventually poor quality of life.

Physicians should be alerted to the potential association between fibromyalgia and migraine that may be linked to its psychological burden.

## Data Availability

The raw data supporting the conclusions of this article will be made available by the authors, without undue reservation.
